# Unbalance Response Analysis and Experimental Validation of an Ultra High Speed Motor-Generator for Microturbine Generators Considering Balancing

**DOI:** 10.3390/s140916117

**Published:** 2014-08-29

**Authors:** Do-Kwan Hong, Dae-Suk Joo, Byung-Chul Woo, Dae-Hyun Koo, Chan-Woo Ahn

**Affiliations:** 1 Electric Motor Research Center, Korea Electrotechnology Research Institute, Boolmosangil 70, Changwon 641-120, Korea; E-Mails: june@keri.re.kr (D.-S.J.); bcwoo@keri.re.kr (B.-C.W.); dhk371@keri.re.kr (D.-H.K.); 2 Department of Mechanical Engineering, Dong-A University, Saha-gu, Busan 604-714, Korea; E-Mail: cwahn@dau.ac.kr

**Keywords:** motor-generator for micro turbine, unbalance response analysis, orbit diagram, critical speed, with and without balancing, rotor dynamics

## Abstract

The objective of the present study was to deal with the rotordynamics of the rotor of an ultra-high speed PM type synchronous motor-generator for a 500 W rated micro gas turbine generator. This paper introduces dynamic analysis, and experiments on the motor-generator. The focus is placed on an analytical approach considering the mechanical dynamic problems. It is essential to deal with dynamic stability at ultra-high speeds. Unbalance response analysis is performed by calculating the unbalance with and without balancing using a balancing machine. Critical speed analysis is performed to determine the operating speed with sufficient separation margin. The unbalance response analysis is compared with the experimental results considering the balancing grade (ISO 1940-1) and predicted vibration displacement with and without balancing. Based on these results, a high-speed motor-generator was successfully developed.

## 1. Introduction

**Figure 1.**
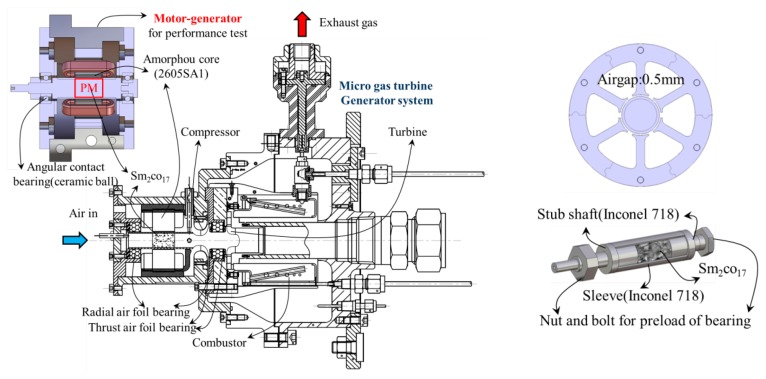
The schematic diagram of a micro gas turbine generator (MTG) system and prototype of the motor-generator.

In the process of developing an unmanned mobile robot, a long lasting power source that has high energy density is essential. High energy density and a large output are simultaneously needed to enable the mobile robots and a robot power suit. In the constant pursuit for new power sources, generators using micro gas turbines are currently being researched in Japan, Belgium, Sweden, and the USA. These devices can provide both components [1]. The Korea Electrotechnology Research Institute (KERI) is developing a motor-generator that fits into a microturbine generator (MTG) system with a power of 500 W. Results from cycle analysis for a projected production of 500 W of output show that the compressor must be in motoring mode with a self-sustaining speed of 200,000 rpm while using 280 W of power. Until the compressor reaches 400,000 rpm, the speed must be continuously increasing, and the compressor should therefore be designed to operate normally and produce an output of 500 W at 400,000 rpm. The aim of the present study is to develop a three-phase high speed and high efficiency synchronous motor. The focus is placed on an analytical approach considering the mechanical dynamic problems. It is essential to deal with dynamic stability at an ultra-high speed. Figure 1 shows the schematic diagram of an MTG system which is supported by air foil bearings and the prototype of a motor-generator (casing and amorphous stator core with winding) which is supported by ceramic ball bearings.

The rotor shape of the motor-generator is considered to connect both rotors by flexible coupling for back-to-back testing. Back-to-back testing was done to evaluate the power generation characteristics of the motor-generator. By using rotordynamic analysis and taking gyroscopic effects into consideration, critical speed and unbalance responses are analyzed. Especially, unbalance response analysis is performed by calculating the unbalance with and without balancing using a balancing machine. The critical speed analysis is performed to determine an operating speed with sufficient separation margin. Moreover, the vibration displacement characteristics are performed by harmonic analysis. Also, the orbit characteristics are calculated and evaluated by transient analysis and experiments using nano-resolution gap sensors. Results by unbalance response analysis are compared with the experimental results considering allowable vibration displacement (API 611) and balancing grade (ISO 1940-1) [2,3].

## 2. Elemental Technology of High Speed Motor

### 2.1. High Speed Motor

High speed and ultra-high speed machines are classified according to velocity and power by Akeshi *et al.* [4]. The power of the motor is expressed by volume, air gap, flux density and the current of the motor as follows:

(1) $$P=k\frac{\pi^{2}}{2}n_{s}D_{i}^{2}L_{c}A_{m}B_{g}$$

where *n_s_*: number of revolutions per second, *D_i_*: stator inner diameter, *L_c_*: rotor length, *A_m_*: line current density, *B_g_*: air gap flux density and k: proportional constant. The power is proportional to volume (*D_i_^2^L_c_*) and velocity [5], so the greater the motor's high speed, the greater the power increase, therefore the volume of the motor can be reduced.

If the power is higher, the rotational speed which will be realized should be reduced due to the limitations of the rotor component materials. On the contrary, the rotational speed will be higher, and the power limitation will be decreased. The limitation between the rotational speed N (krpm) of the high speed motor and output power P (kW) is as follows:

(2) $$N^{3.3}P=6.2\times 10^{6}$$

**Figure 2.**
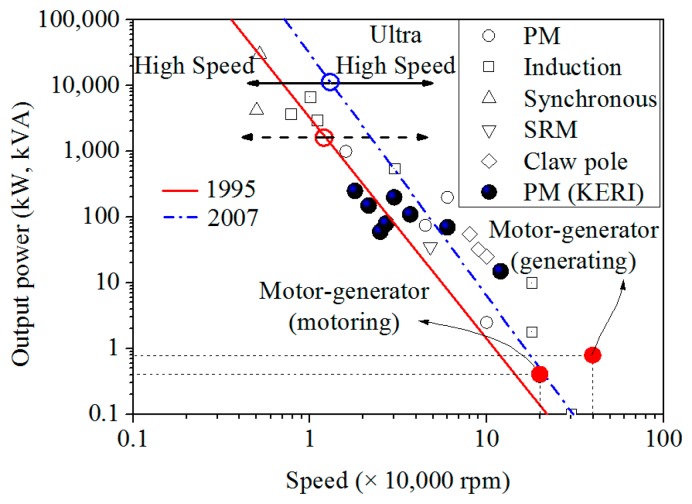
The relation of rotational speed and output power.

In Equation (2), the result was derived from an induction motor in 1995 [4]. High speed and ultra-high speed are not definitively divided and classified by the left area of the line (high speed) and the right area of the line (ultra-high speed). Figure 2 shows that PM machines are particularly prevalent among small high speed machines [6–13]. The relationship *N^3.3^P* = 6.2 × 10^6^, is indicated by a solid line in the diagram below.

Based on the released induction motor and the permanent magnet motor, high speed and ultra-high speed machines were also categorized according to velocity and power by Binder and Schneider from 1995 to 2006 [6]. The relation between rated powers and speeds in the diagram is in good agreement with the correlation empirically found by Binder and Schneider who obtained the relationship log*f* = 4.27 − 0.275log*P* (consequently, *P*∼1/*f^3.6^*), which is presented with a dash-dot line in a diagram [6,7]. As a result, the motoring mode of the motor-generator is in the ultra-high speed area, but as a result of the latter, the motoring mode of the motor-generator is around the high speed boundary. Much research and development has been done in this area in order to move the limiting line to the upper and right side as shown in Figure 2.

### 2.2. The Effects on Magnetic Field

**Figure 3.**
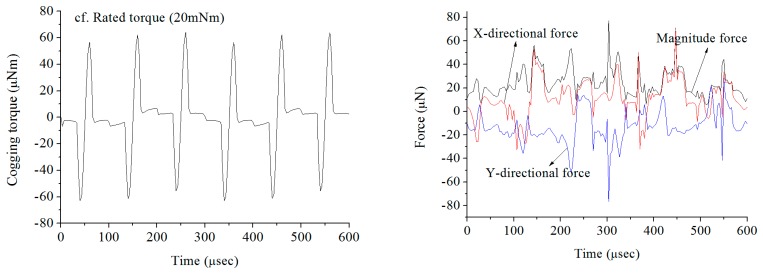
Cogging torque and force of rotor (magnet + sleeve).

Figure 3 represents the cogging torque and force of the rotor (magnet + sleeve). The amplitude of the cogging torque is trivial. Also, the rotor (magnet + sleeve) has very small amounts of force. The effects on the magnetic field are examined in the operation of a high-speed machine under miniaturization. However, the cogging torque is trivial considering the rated torque and the generated force of the rotor (magnet + sleeve) is very small. Therefore, the magnetic field effects are totally ignored and considered as only mechanical lumped parameters.

### 2.3. Backward/Forward Whirling Due to Unbalance Forces

The equations that need to be solved in order to obtain the response (q) to unbalance forces are outlined. The equation of motion is given by:

(3) $$\text{M}\ddot{\text{q}}+\text{G}\dot{\text{q}}+\text{kq}=\text{f}$$

where: $\text{M}=\left[\begin{array}{cc} \text{M} & 0\\ 0 & \text{M} \end{array}\right]\text{G}=\left[\begin{array}{cc} 0 & \text{G}\\ -\text{G} & 0 \end{array}\right]\text{K}=\left[\begin{array}{cc} {\text{K}}_{\text{x}} & 0\\ 0 & {\text{K}}_{\text{y}} \end{array}\right]$ M: mass matrix, G: gyroscopic matrix, K: stiffness matrix

The unbalance forces are co-rotating at speed Ω and are equal to:

$$\text{f}=\left\lfloor \begin{array}{cc} {\text{f}}_{\text{x}} & {\text{f}}_{\text{y}} \end{array}\right\rfloor \;{\text{f}}_{\text{x}}=\left[\begin{array}{c} \text{e}\Omega^{2}\\ 0 \end{array}\right]\cos\Omega \text{t}{\;\text{f}}_{\text{y}}=\left[\begin{array}{c} 0\\ \text{e}\Omega^{2} \end{array}\right]\sin\Omega \text{t}$$

define: q = q_c_ cosΩt + q_s_ sin Ωt:

(4) $$\left[\begin{array}{cc} \text{K}-\Omega^{2}\text{M} & \Omega \text{G}\\ -\Omega \text{G} & \text{K}-\Omega^{2}\text{M} \end{array}\right]\left(\begin{array}{c} {\text{q}}_{\text{c}}\\ {\text{q}}_{\text{s}} \end{array}\right)=\left(\begin{array}{c} \left(\begin{array}{c} \text{e}\Omega^{2}\\ 0 \end{array}\right)\\ \left(\begin{array}{c} 0\\ \text{e}\Omega^{2} \end{array}\right) \end{array}\right)$$

define: p = q_c_ + iq_s_:

We can write: $(\text{K}-\Omega^{2}\text{M})\text{p}-\text{i}\Omega \text{G}\bar{\text{p}}=\Omega^{2}\left(\begin{array}{c} \text{e}\\ \text{ie} \end{array}\right)$

(5) $$\text{p}={\text{p}}_{\text{f}}{\text{e}}^{\text{i}\Omega \text{t}}+{\text{p}}_{\text{b}}{\text{e}}^{-\text{i}\Omega \text{t}}$$

and substituting in the equation of motion:

(6) $$\text{M}\ddot{\text{x}}+\Omega \text{G}\dot{\text{y}}+{\text{k}}_{\text{x}}\text{x}=\Omega^{2}\text{e}\;\cos\Omega \text{t}$$

(7) $$\text{M}\ddot{\text{y}}-\Omega \text{G}\dot{\text{x}}+{\text{k}}_{\text{y}}\text{y}=\Omega^{2}\text{e}\;\sin\Omega \text{t}$$

Let p = x + iy, we have then from Equations (6) and (7):

(8) $$\text{M}\ddot{\text{p}}-\text{i}\Omega \text{G}\dot{\text{p}}+\frac{1}{2}({\text{k}}_{\text{x}}+{\text{k}}_{\text{y}})\text{p}+\frac{1}{2}({\text{k}}_{\text{x}}-{\text{k}}_{\text{y}})\bar{\text{p}}=\Omega^{2}{\text{ee}}^{\text{i}\Omega \text{t}}$$

Introduce Equation (5) into Equation (8):

$$\left[\begin{array}{cc} {\text{K}}_{\text{m}}-\Omega^{2}(\text{M}-\text{G}) & {\text{K}}_{\text{d}}\\ {\text{K}}_{\text{d}} & {\text{K}}_{\text{m}}-\Omega^{2}(\text{M}+\text{G}) \end{array}\right]\left(\begin{array}{l} {\text{p}}_{\text{f}}\\ {\bar{\text{p}}}_{\text{b}} \end{array}\right)=\left(\begin{array}{c} \Omega^{2}{\text{u}}_{\text{b}}\\ 0 \end{array}\right)$$

where ${\text{K}}_{\text{m}}=\frac{1}{2}({\text{K}}_{\text{x}}+{\text{K}}_{\text{y}}),{\text{K}}_{\text{d}}=\frac{1}{2}({\text{K}}_{\text{x}}-{\text{K}}_{\text{y}})$

## 3. Rotordynamic Analysis and Experimental Validation

### 3.1. Ultra High Speed Motor-Generator

**Figure 4.**
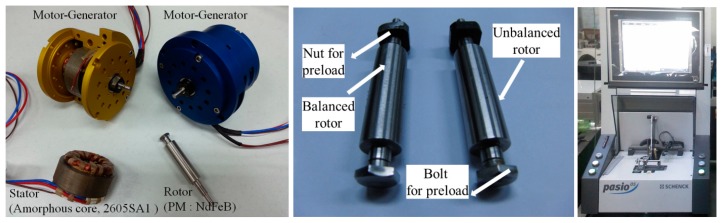
Motor-generator, unbalanced rotor, balanced rotor and balancing machine.

Figure 4 shows the configuration of the permanent magnet synchronous motor for an ultra-high speed motor-generator for a microturbine generator system. The rotor is manufactured by press fitting and supported by an angular contact bearing preloaded on the inner race. A bolt and nut are utilized to preload the inner race. The balancing of the rotor consisted of a bolt and nut and is essential to operate at high speed.

### 3.2. Balancing by Balancing Machine

Balancing is the process of aligning a principal inertia axis with the geometric axis of rotation through the addition and removal of material. By balancing, the centrifugal force is reduced, minimizing vibration, noise and associated wear. The balancing of the developed rotor is performed by Pasio 05 system, made by a Schenck firm as shown in Figure 3. The unbalance of the nut is measured as 0.655 g·mm (G = 300) and the bolt is 0.26 g·mm (G = 120) by two planes balancing performance before balancing. The unbalance of the nut is measured as 0.0141 g·mm (G = 6.33) and the bolt is 0.0137 g·mm (G = 6.33) by the balancing performance of two planes after balancing.

### 3.3. Modal Testing

The natural frequency analysis of the rotor is validated by experimental results. The experimental natural frequency and mode shape of the tested rotor with free-free boundary conditions is used to update the free-free finite element representations of the same.

**Figure 5.**
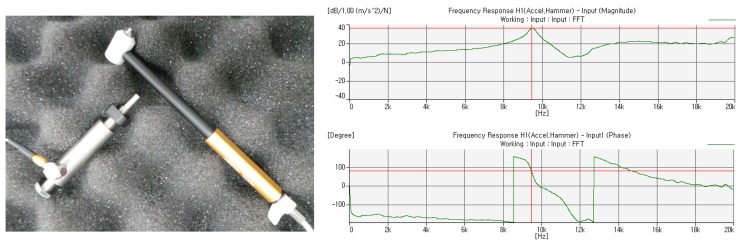
Modal testing result: prototype (left) FRF (upper) phase (lower).

The FEM result (fundamental natural frequency 9254 Hz) is in good accord with that of modal testing (fundamental natural frequency 9459 Hz) with a maximum 3.5% error. Figure 5 shows the rotor prototype model, the amplitude of the frequency response function (FRF) and the phase of the FRF.

### 3.4. Critical Speed

**Figure 6.**
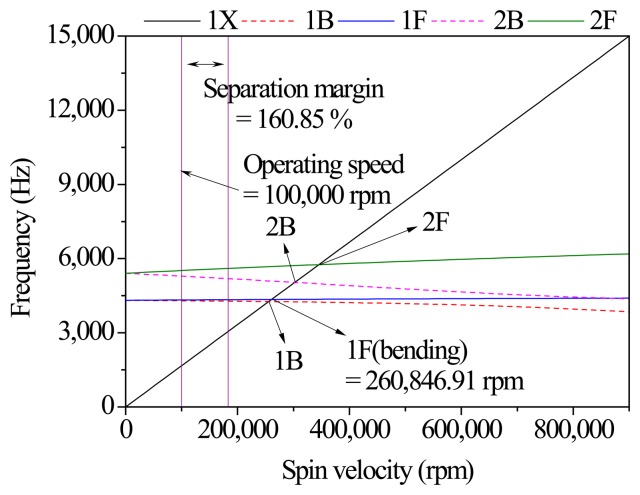
Critical speed of the motor-generator.

The critical speed of the rotor considering the rotation and gyroscopic effect should be above the operating speed (100,000 rpm), and have a sufficient separation margin, 161%, as shown in Figure 6. The 1st forward whirling critical speed (260,847 rpm) of the developed model is higher than the operating speed (100,000 rpm). An appropriate separation margin is typically 20%∼30% between the operating speed and the bending forward whirling critical speed.

### 3.5. Unbalance and Allowable Vibration Displacement (API 611)

The unbalance vibration response analysis of the rotor which was considered balanced enabled the prediction of the expected vibration amplitude by unbalance at high speed. The calculated unbalance is as shown below [2,3]:

(9) $${\text{U}}_{\max}=9549\times \frac{\text{G}\times \text{M}}{\text{N}}(\text{g}•\text{mm})$$

where G is G grade (303, 120, 6.33), M is the weight of the rotor (0.04523 kg), N is the rotational speed (100,000 rpm), U_max_ (U) is the unbalance. The calculated unbalance is applied to the unbalanced rotor and balanced rotor once and ten times, respectively, in this paper [11]. Generally, from quadruple to ten times the calculated unbalance can be applied to the rotor. Despite the fact that it was ten times greater than the calculated unbalance magnitude, the maximum vibration displacement around the bearing was within the allowable vibration displacement, API 611, so the dynamic stability has no problems. The allowable vibration for a rotating machine is evaluated by API 611 as below [2]:

(10) $$\text{A}=25.4\sqrt{\frac{12,000}{\text{N}}}(\mu {\text{m}}_{\text{p}-\text{p}},\text{API}\;\text{Standard}611)$$

where A is the allowable vibration displacement, and N is the rotational speed. The unbalance vibration responses around the bearing supporting position are satisfactory with allowable vibration displacement (8.8 μm_p-p_).

### 3.6. Unbalance Response Analysis Considering Balancing

**Figure 7.**
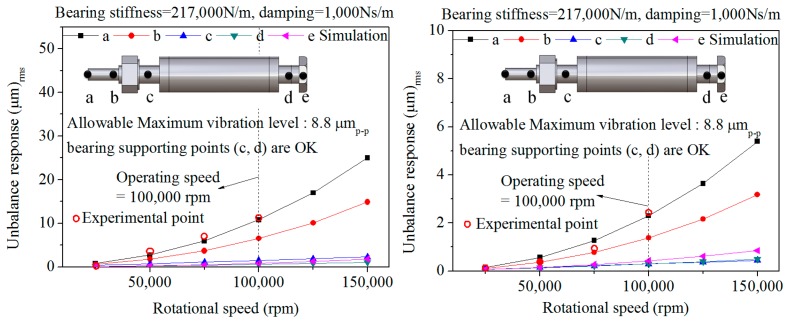
The comparison between experiment and simulation by unbalance response analysis: Unbalanced rotor (left, G303, G120, U_max_) balanced rotor (right, G6.33, 10U_max_).

**Figure 8.**
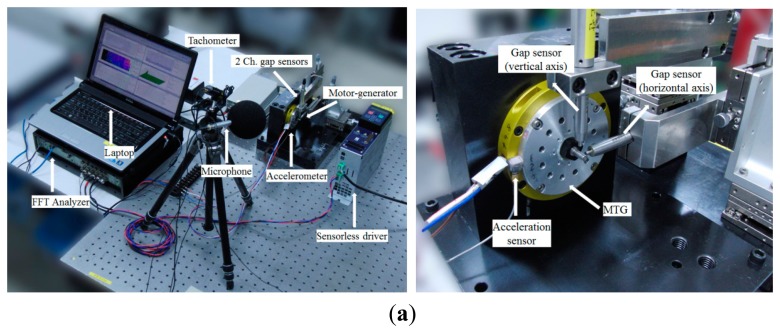

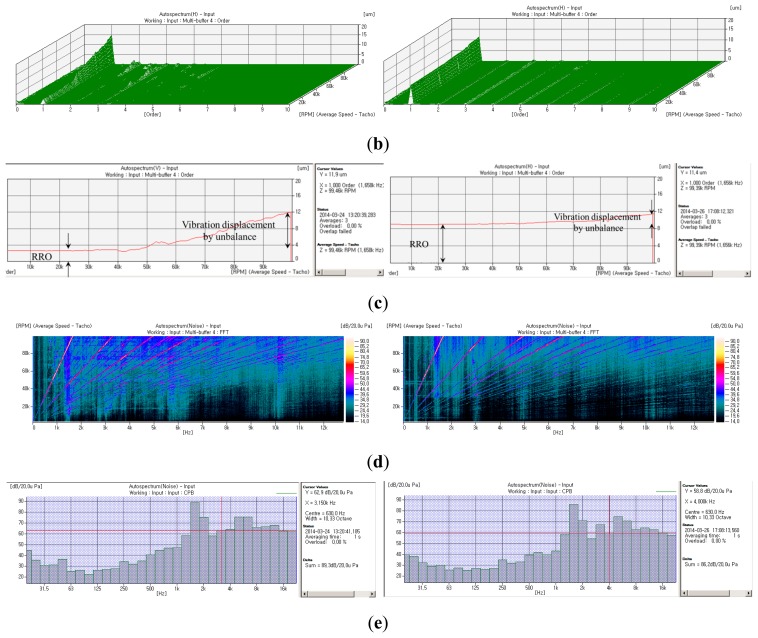
Operating test results: Unbalanced rotor (left) balanced rotor (right). (**a**) Test setup for experimental validation (**b**) Horizontal vibration displacement of rotor (**c**) Horizontal vibration displacement of rotor (1 order) by order analysis (**d**) Noise evaluation according to operating speed (**e**) 1/3 octave evaluation @ 100,000 rpm.

Figure 7 shows the comparison between experiment and analysis. In the case of the unbalanced rotor model, the unbalance data before balancing is applied to the nut and bolt to provide the preload for the angular contact ceramic ball bearing. Also, in case the of the balanced rotor model, the unbalance sheet by two planes balancing is applied to the same parts. The experiment results which contain the initial Repeatable Run Out (RRO) are utilized in Figure 8c. The main RRO is caused by the bent end of the rotor by the nut and bolt parts to the preloaded angular contact ceramic ball bearing in the elastic region. The analysis results are in accord with the experiment results.

### 3.7. Experimental Validation

Experimental validation is performed to verify the analysis result. Figure 8 shows the setup for the orbit measurement of the balanced rotor and unbalanced rotor.

Two channel gap sensors and amplifiers are utilized. Also a tachometer, accelerometer and microphone are utilized to evaluate noise and vibration using Fast Fourier Transform (FFT) and octave analysis. Gap sensors with nano-level resolution are installed horizontally and vertically at a 90° interval. The rotational speed runs up to the rated speed (100,000 rpm) by a 1 kW rated sensorless inverter. The gap sensors are measured by a tachometer trigger. Figure 8 represents the operating test results. Figure 8b shows and compares the horizontal vibration displacement between the balanced rotor and unbalanced one. In the case of an unbalanced rotor, the higher the rotational speed, the more the horizontal vibration displacement which will be realized is increased due to the unbalance of the rotor. On the other hand, the higher the rotational speed, the more horizontal vibration displacement which will be realized is gently increased in the case of the balanced rotor. This means that balancing of the rotor is achieved well. It is assumed that there is assembly tolerance, foundation, vibration caused by electrical problems, natural frequency differences and bending of the rotor, *etc.* The thin end of the rotor to fix the flexible coupling was bent by the nut and bolt parts to preload the angular contact ceramic ball bearing. Figure 8b represents the order analysis results which contain harmonic order. Figure 8c shows the first order of horizontal vibration displacement which has the initial RRO of those rotors respectively. Figure 8d represents noise evaluation which contains a similar result. Figure 8e shows the sound pressure level (SPL) of the balanced rotor is smaller than that of the unbalanced rotor by 1/3 octave analysis. The balanced rotor is quieter than the unbalanced rotor by about 3 dB (A).

## 4. Conclusions

The purpose of the present study was to introduce the dynamic analysis and experiments at the heart of a 500 W rated MTG system, the motor-generator. To develop a three-phase high speed and high efficiency synchronous motor, the focus is placed on an analytical approach considering the mechanical dynamic problems. It is essential to deal with dynamic stability at ultra-high speeds. Unbalance response analysis is performed by calculating the unbalance with and without balancing by a balancing machine. The balanced rotor is better than the unbalanced rotor in terms of the vibration displacement of rotor, and noise and so on. Based on these results, a high speed motor-generator is successfully developed.
